# The Detection of Gait Events Based on Smartphones and Deep Learning

**DOI:** 10.3390/bioengineering12050491

**Published:** 2025-05-04

**Authors:** Kaiyue Xu, Wenqiang Yu, Shui Yu, Minghui Zheng, Hao Zhang

**Affiliations:** 1 College of Mechanical Engineering, Shandong Huayu University of Technology, Dezhou 253034, China; jjxukaiyue@163.com (K.X.); zhengminghui1987@126.com (M.Z.); 2 College of Information Engineering, Dalian University, Dalian 116622, China; 3 School of Physical Science and Technology, Southwest Jiaotong University, Chengdu 610031, China; yushui1206@163.com

**Keywords:** gait analysis, gait event, smartphone, deep learning, mobile health

## Abstract

This study aims to detect gait events using a smartphone combined with deep learning and evaluate the remote effects and clinical significance of this method in different elderly populations and patients with cerebral small vessel disease (CSVD). In total, 150 healthy individuals aged 20–70 years were asked to attach a smartphone to their thighs and walk six gait cycles at self-selected low, normal, and high speeds, using an insole pressure sensor as the reference standard for gait events. A deep learning model was then established using BiTCN-BiGRU-CrossAttention, and two models (TCN-GRU and BiTCN-BiGRU) were compared. In total, 48 elderly (25 healthy, 12 with mild cognitive impairment, 11 with Parkinson’s disease) participated in an online home assessment, completing single-task and cognitive dual-task walking. Overall, 35 CSVD patients participated in an offline clinical assessment, completing single-task, cognitive dual-task, and physical dual-task walking. The BiTCN-BiGRU-CrossAttention model had the lowest MAE for detecting gait events compared to the other models. All models had lower MAEs for detecting heel strikes than toe-offs, and the MAE for low and high walking was higher than for normal speed walking. There were significant differences (*p* < 0.05) in gait parameters (Cadence, Stride time, Stance phase, Swing phase, Stance time, Swing time, Stride length, and walking speed) between single-task and cognitive dual-task walking for all online elderly participants. CSVD patients showed significant differences (*p* < 0.05) in gait parameters (Cadence, Stride time, Stance phase, Swing phase, Stance time, Stride length, and walking speed) between single-task and cognitive dual-task and between single-task and physical dual-task walking.

## 1. Introduction

Gait analysis relies on accurately determining events occurring during walking, such as heel strike and toe-off. Identifying these events allows for examining parameters such as Stance phase, Swing phase, Stride time, and Stride length [1]. Traditional methods, such as optical motion capture systems, force plates, and electromyography [2], can provide professional results but typically require substantial costs and support personnel [3], making them inaccessible to many users.

Furthermore, with the rapid development of smartphones, their application in the health field is becoming increasingly widespread, particularly in motion monitoring. Research has shown that built-in smartphone sensors can measure and quantify human movement [4]. Additionally, several studies have demonstrated the effective application of smartphone built-in accelerometers, gyroscopes, and other sensors in gait analysis, covering various populations such as the elderly [5] and Parkinson’s disease patients [6]. Smartphones provide a portable and inexpensive method that can be applied anywhere, not limited to specific settings. Many studies have validated the smartphone against standard equipment [4,7,8,9,10,11,12], but some studies have only included a small number of young, healthy individuals [4,7,8,9,10] analyzed a relatively limited range of gait parameters [8,9,10], did not mention the impact of different walking speeds on the results [4,8,10,11,12], and did not evaluate the application of the method in daily home environments [4,7,8,9,11,12]. Moreover, these studies only reported consistency compared to standard equipment and did not mention the time error in detecting gait events [4,7,8,9,10,11,12]. Therefore, this study aims to explore a method using a smartphone and deep learning to detect gait events at different walking speeds, with the main highlights as follows:

Gait signals represent a macroscopic manifestation of neuronal network activity involving the coordinated regulation of central pattern generators (CPGs). The mechanisms of information transmission and processing within neuronal networks can be simulated using mathematical models. Studies have shown that periodic spiking activity in unidirectionally coupled Hindmarsh–Rose neuronal chains can induce novel slow rhythms, transitioning from chaotic to regular dynamics along the chain [13]. This phenomenon bears similarity to the gait rhythms driven by CPGs, providing theoretical support for understanding the generation mechanisms of complex gait signals and for employing deep learning methods to identify gait events. Consequently, this study focuses on leveraging built-in smartphone sensors combined with deep learning to detect gait events from smartphone signals in a controlled environment, aiming to provide a portable, low-cost solution for human behavior monitoring.

(1) This study is the first to employ built-in smartphone sensors combined with deep learning to detect gait events, providing a novel approach for portable gait analysis.
(2) The proposed model in this study applies to different walking speeds, and the detection time errors of different deep learning models are compared.
(3) The external application effects of this method are remotely assessed in different elderly populations in daily home environments.
(4) The clinical significance of this method is evaluated in a population with cerebral small vessel disease.

## 2. Materials and Methods

### 2.1. Software Platform

Given the widespread user base of the WeChat platform, WeChat mini programs offer a convenient and lightweight user experience without the need for downloading and installation. Moreover, WeChat mini programs support cross-platform operations, making them easy to promote [14]. Therefore, this study developed a WeChat mini program as a software tool for gait analysis and successfully published it on the WeChat platform.

### 2.2. Participants

The participants in this study were divided into offline and online participants. Table 1 summarizes the demographic details of the participants.

Health: This study recruited 150 healthy individuals offline. The inclusion criteria are as follows: (1) age greater than 20 years; (2) ability to walk continuously for at least 10 m without assistance from others or walking aids. The exclusion criteria are as follows: the presence of mental, neurological, or physical impairments and uncorrectable visual impairments.

Elderly: This study screened 48 older adults from WeChat mini program users as online participants. Among them, 25 older adults were in good physical condition, 12 had mild cognitive impairment, and 11 had Parkinson’s disease.

CSVD: This study recruited 34 patients with cerebral small vessel disease (CSVD) from the Department of Neurology at the Affiliated Zhongshan Hospital of Dalian University offline. All patients underwent head magnetic resonance imaging (MRI) examinations. The Fazekas scoring system was used to grade the burden of white matter lesions [15]. The inclusion criteria are as follows: Fazekas score ≥ 1; confirming the presence of CSVD. The exclusion criteria are as follows: severe brain diseases; mental disorders; cognitive impairments; and physical disabilities that affect the examination.

The Research Project Ethics Review Committee of the Affiliated Zhongshan Hospital of Dalian University approved this study. All offline participants provided written informed consent, and all online participants read the user guide and agreed to collect personal information in advance within the WeChat mini program.

### 2.3. Experimental Design

**Figure 1:**
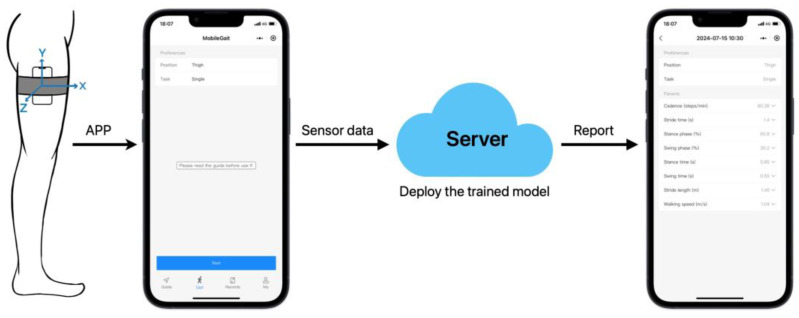
System architecture of the WeChat mini program.

Health: Previous studies have shown that sensors that are in closer proximity to the foot-ground contact point are facilitated in gait event detection [16]. Considering the weight of smartphones, this study prioritized the thigh position for data collection. As shown in Figure 1, a smartphone (iPhone 13) was attached to the thigh of the participants using a belt-like Velcro strap, while an insole pressure sensor (M3232L, Roxifsr, China; Sampling rate: 50 Hz) was used as the reference standard for gait events. Each participant was asked to walk in a straight line for 6 gait cycles at self-selected [17] normal, low, and high speeds in a horizontal corridor.

Elderly: Online participants completed 2 rounds of home-based gait assessment trials. The first round was a single-task walking (STW) trial, i.e., normal walking. The second round was a cognitive dual-task walking (Verbal Fluency Test, VFT) trial; participants were asked to name the fruits or animals they knew while walking.

CSVD: Patients completed three rounds of gait trials in a horizontal corridor. The first round was STW. The second round was a VFT. The third round was a physical dual-task walking (PTW) trial, which required patients to carry a tray with a water bottle with both hands to keep it from tipping over during walking.

## 3. Experiments and Results

### 3.1. Data Collection and Processing

**Figure 2:**
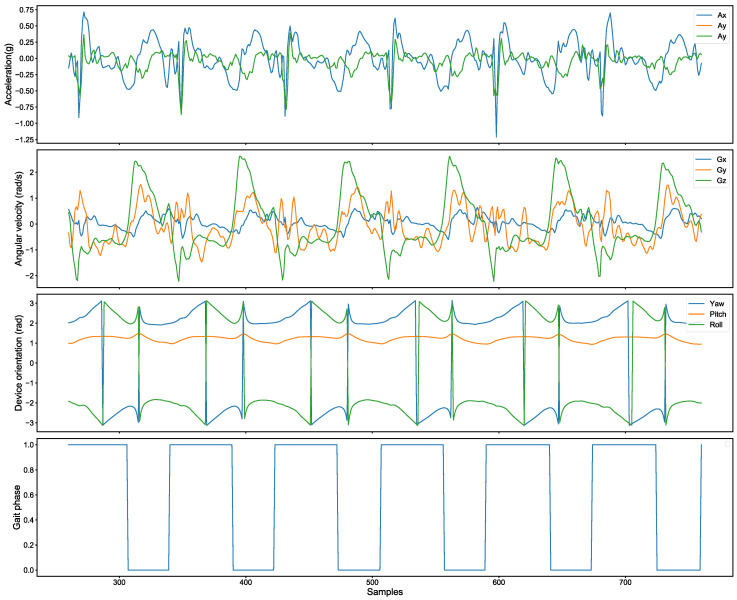
The smartphone collects raw sensor data and the truth label (1 for the Stance phase; 0 for the Swing phase).

The smartphone captures triaxial acceleration data (Ax,Ay,Az) and triaxial angular velocity data (Gx,Gy,Gz) at a sampling rate of 50 Hz, as well as the smartphone’s rotation angles (Yaw,Pitch,Roll) around the ZXY axes in three-dimensional space, as depicted in Figure 2. During the smartphone data collection phase, the raw sensor data may be affected by noise due to factors such as body tremors and device deviations. Kalman filtering and low-pass filters are used to remove noise. When walking, the overall variation in acceleration Az, angular velocity Gx, and rotation angle Pitch was relatively small; these three features were excluded.

### 3.2. Building Dataset

Input–output data pairs are constructed using a sliding window approach [18]. The input X represents the sensor data collected by the smartphone, and the output Y represents the corresponding reference gait events for X. The first input–output data pair is $X_{1}$, $X_{2}$, …, $X_{\omega }$ and $Y_{\omega +1}$; the t input–output data pair is $X_{t}$, $X_{t+1}$, …, $X_{\omega +1}$ and $Y_{\omega +t+1}$; and the last input–output data pair is $X_{n-\omega }$, $X_{n-\omega +1}$, …, $X_{n-1}$ and $Y_{n}$, where ω is the window length (ω= 40). From the 150 healthy individuals aged 20–70 years across six age groups, the input–output data pairs from participants in each age group were divided into 60% for the training set, 20% for the validation set, and 20% for the test set.

### 3.3. Deep Learning Models

**Figure 3:**
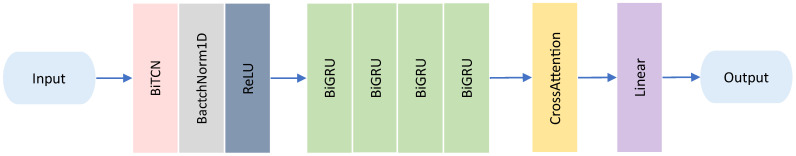
The structure of BiTCN-BiGRU-CrossAttention.

This study proposes a BiTCN-BiGRU-CrossAttention model that integrates the temporal convolutional properties of TCN, the bidirectional temporal modeling capabilities of BiGRU, and the key feature focusing ability of CrossAttention. Specifically, TCN extracts local features from time series data through multi-layer residual connections; BiGRU captures long-term dependencies via forward and backward gating mechanisms; and CrossAttention enhances the identification of critical gait events through attention weights (Figure 3). This study also compares two different models: TCN-GRU and BiTCN-BiGRU.

All the models use the Adam optimizer and Mean Absolute Error as the loss function. The best model is determined by minimizing the validation set error by training for 50 epochs.

### 3.4. Error Measurement

**Figure 4:**
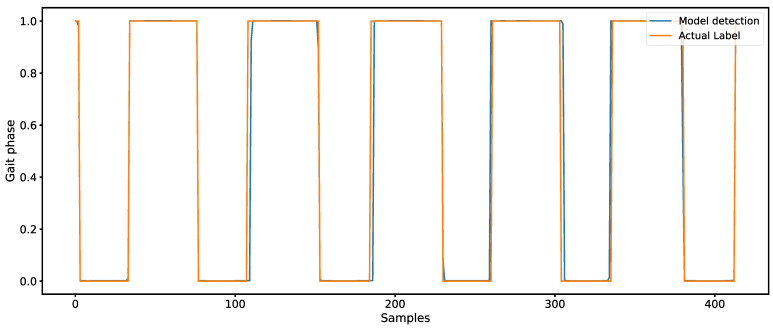
The output of the BiTCN-BiGRU-CrossAttention model and the truth label.

The time difference between the gait events detected by the model and the reference standard gait events, i.e., the Mean Absolute Error (MAE, unit: milliseconds), was used as an evaluation metric for model performance on the test set. A smaller time difference indicates a higher model accuracy in detecting gait events. Figure 4 shows an example of the output of the BiTCN-BiGRU-CrossAttention model and the truth label.

### 3.5. Calculation of Gait Parameters

**Figure 5:**
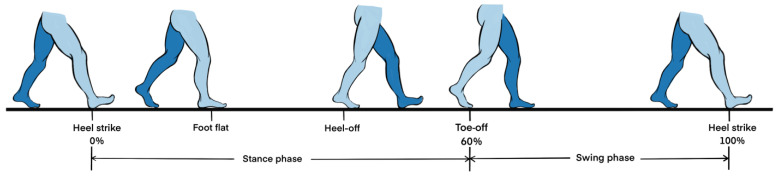
The phases of the gait cycle.

For online elderly participants and CSVD patients, spatiotemporal gait parameters were calculated based on gait events detected by the best deep-learning model. To accurately estimate walking distance at different walking speeds, this study adopted a linear regression model based on walking speed to achieve an adaptive estimation of walking distance [19]. Figure 5 is a schematic diagram of a gait cycle.

### 3.6. Result

**Figure 6:**
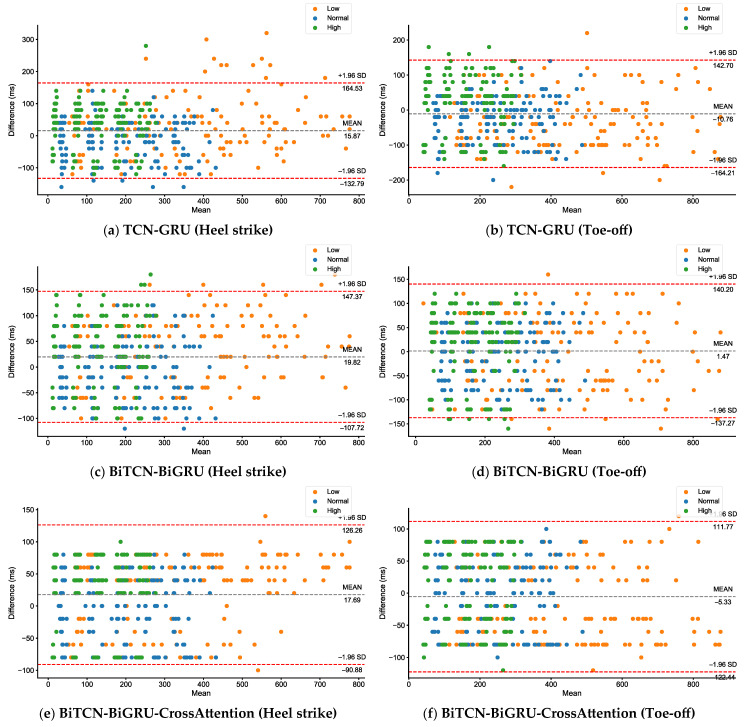
Bland–Altman plots show the difference (model detection—truth label) between various walking speeds (low, normal, and high). The dashed gray line represents the overall mean difference across all conditions, while the dashed red lines provide the limits of agreement (±1.96 SD) based on the pooled data.

Table 2 shows the MAE (Mean ± SD) of heel strike and toe-off detection using all models at different walking speeds for healthy participants. The results indicate that the BiTCN-BiGRU-CrossAttention model has lower MAEs when detecting heel strike and toe-off at all three walking speeds compared to the other models. Furthermore, all models have a lower MAE when detecting heel strike compared to toe-off; the MAE at normal speed is lower than at low and high speeds Figure 6.

Table 3 shows significant differences (*p* < 0.05) in gait parameters (Cadence, Stride time, Stance phase, Swing phase, Stance time, Swing time, Stride length, and walking speed) between the STW and VFT conditions for online elderly participants. For CSVD patients, significant differences (*p* < 0.05) in gait parameters (Cadence, Stride time, Stance phase, Swing phase, Stance time, Stride length, and walking speed) existed between the STW and VFT conditions, as well as between the STW and PTW conditions.

## 4. Discussion

This study addresses gait discovery from smartphone signals in the context of intelligent detection and the control of human behavior. The experimental results demonstrate that the proposed method exhibits good reliability and validity, particularly at normal walking speeds. Additionally, the method effectively detects changes in gait parameters under dual-task conditions in different groups of older adults and CSVD patients. This study addresses how to achieve gait event detection using low-cost devices combined with deep learning.

Similar studies have used a single commercial IMU or smartphone to detect gait events and report the time error. Fadillioglu et al. proposed a gait events detection method using a gyroscope attached to the right shank with a rule-based algorithm, reporting an MAE of 11 ± 3 ms and 29 ± 11 ms for heel strike and toe-off [20]. Gonzalez et al. presented a gait events detection method using an IMU attached at the waist; the lowest MAE for heel strike and toe-off events using a rule-based method was 15 ms and 9 ms [21]. McCamley et al. proposed a gait events estimation method based on Gaussian CWT using an accelerometer on the waist, with an MAE of 19 ms and 32 ms for heel strike and toe-off [22], respectively. Arshad et al. used a single waist-worn sensor with a CNN-BiGRU-SelfAttention deep learning model, achieving an MAE of 6.239 ms and 5.24 ms for heel strike and toe-off event predictions, respectively. To our knowledge, one study used a smartphone attached at the lower back and hip, employing three different heel strike event detection methods using acceleration data, with errors for heel strike recorded at 0.012 ± 0.056 s, 0.005 ± 0.051 s, and 0.005 ± 0.050 s; however, these results were limited to a small sample of 11 young, healthy individuals and did not mention toe-off moment errors or the influence of different walking speeds on gait event detection [23]. Although the MAE obtained by the model proposed in this paper are not as low as those in previous studies, they are still within an acceptable range. The data quality acquired by built-in smartphone sensors is difficult to compare with commercial IMUs. Built-in smartphone sensors are prone to accumulating more noise at low speeds. At high speeds, there are higher requirements for the sampling rate. The WeChat mini program platform limits the maximum sampling rate of sensors, such as accelerometers and gyroscopes, to 50 Hz, but high-sampling-rate smartphone sensors can record data more frequently and capture rapidly changing motions more accurately, which may introduce more noise [24].

In online elderly participants and offline CSVD patients, gait parameters were negatively affected by the addition of dual tasks, which may have implications for assessing older adults, as the addition of dual tasks may expose deficits not observed in single-task assessments [25]. The finding that individuals with cognitive decline exhibit gait impairments, particularly under dual-task conditions, may be explained by the neuropathological changes in specific brain regions involved in motor planning and execution, which occur in the early stages of dementia [26]. Patients with Parkinson’s disease are affected by dual-task walking as spatiotemporal gait parameters deteriorate when walking is combined with a secondary task [27]; patients may experience symptoms such as freezing [28] and falling [29]. CSVD is associated with cognitive [30] and motor impairments [31,32], and patients may not have overt clinical symptoms [33,34]. However, CSVD patients have lower gait speed [35] and are likelier to exhibit abnormal gait characteristics [36] under dual-task activities. Therefore, quantitative gait analysis using a smartphone can detect changes in gait parameters in the early stages of cognitive decline and patients with neurodegenerative diseases, serving as a non-invasive biomarker for disease detection and enabling the timely implementation of targeted interventions.

Compared to existing studies, this research is the first to propose a method for detecting gait events using built-in smartphone sensors combined with deep learning, validating the model’s adaptability across different walking speeds, and achieving remote gait assessment in home environments through a software application without incurring additional hardware costs, thereby addressing a gap in the field’s practical applications. Notably, the BiTCN-BiGRU-CrossAttention model employed in this study represents a novel deep learning approach, distinguished by its integration of TCN, BiGRU, and CrossAttention. This model effectively extracts both local and global temporal features from gait signals, demonstrating good adaptability in multi-speed gait detection compared to traditional methods, such as rule-based algorithms or single neural network architectures. Future improvements should focus on optimizing the weight distribution of the attention mechanism to enhance model performance further.

The present study has several limitations. This study constructed a dataset for detecting gait events using deep learning, encompassing participants aged 20 to 70 years across different age groups. However, the dataset primarily included healthy individuals, which may not fully capture the gait characteristics of populations with neurological or motor function impairments. Furthermore, although the gender distribution within the healthy cohort’s age groups was relatively balanced, the age distribution may not entirely represent older populations susceptible to gait-related disorders, such as Parkinson’s disease or cerebral small vessel disease. This could lead to class imbalance issues in the dataset, potentially affecting the model’s performance and generalizability. Class imbalance is a common challenge in machine learning and deep learning research, and non-representative datasets or overly complex model configurations may introduce bias in the results [37]. The study’s gait trial environment was limited to level straight-line walking, which can mostly meet gait analysis needs. However, walking environments in daily life have uncertainties, such as uneven surfaces, turns, and avoidance. Future research should further expand the types of participants, covering more types of patients with neurological diseases or motor impairments.

## 5. Conclusions

This study combines smartphone built-in sensors and deep learning to detect gait events. The experimental results show that the BiTCN-BiGRU-CrossAttention model demonstrates higher accuracy in detecting heel strike and toe-off, especially at normal walking speeds. Furthermore, this method can effectively differentiate gait differences between healthy older adults, individuals with mild cognitive impairment, Parkinson’s disease, and cerebral small vessel disease patients during single-task and dual-task walking. The smartphone-based gait analysis method proposed in this study is easy to operate, has a low cost, and promotes detection, showing promise for applications in remote rehabilitation management, clinical assessment, and other fields.

## Figures and Tables

**Table 1:** Summary of participants.

Participant	Feature	Age Range					
		20~29	30~39	40~49	50~59	60~69	70~79
Health (N = 150)	Male	11	14	13	16	14	13
	Female	11	11	12	13	12	10
	Age (years)	25.55 ± 2.60	35.40 ± 2.65	45.08 ± 2.50	55.21 ± 2.61	64.65 ± 2.71	73.96 ± 2.33
	Height (cm)	171.55 ± 8.78	169.00 ± 6.23	169.40 ± 8.36	169.38 ± 7.52	167.85 ± 9.57	166.37 ± 9.41
	Weight (kg)	63.80 ± 9.50	64.96 ± 6.81	64.86 ± 6.57	63.50 ± 6.03	64.65 ± 2.71	62.96 ± 7.35
Elderly (N = 48)	Male	-	-	-	-	6	20
	Female	-	-	-	-	10	12
	Age (years)	-	-	-	-	64.25 ± 2.79	75.31 ± 3.21
	Height (cm)	-	-	-	-	163.75 ± 7.03	165.25 ± 7.49
	Weight (kg)	-	-	-	-	67.31 ± 12.82	63.97 ± 9.37
CSVD (N = 34)	Male	-	-	-	1	7	16
	Female	-	-	-	1	3	6
	Age (years)	-	-	-	58.50 ± 0.71	64.10 ± 2.69	74.91 ± 2.69
	Height (cm)	-	-	-	167.00 ± 7.07	171.00 ± 7.90	169.96 ± 8.22
	Weight (kg)	-	-	-	70.00 ± 14.14	69.95 ± 8.19	68.64 ± 8.11

**Table 2:** MAE for the detection of gait events across models and walking speeds.

Gait Events	Speed	Model		
		TCN-GRU	BiTCN-BiGRU	BiTCN-BiGRU-CrossAttention
Heel strike	Normal	47.87 ± 1.99	45.47 ± 1.64	42.53 ± 1.25
	Low	66.80 ± 3.14	62.93 ± 1.86	58.40 ± 1.07
	High	65.20 ± 1.92	61.73 ± 1.99	56.93 ± 1.22
Toe-off	Normal	49.60 ± 2.15	47.73 ± 1.62	45.47 ± 1.45
	Low	73.06 ± 2.28	67.73 ± 1.79	59.20 ± 1.13
	High	72.00 ± 2.14	65.46 ± 1.97	58.27 ± 1.09

**Table 3:** *T*-test comparison between single-task and dual-task walking.

Participant	Task and p	Gait Parameters							
		Cadence (Steps/min)	Stride Time (s)	Stance Phase (%)	Swing Phase (%)	Stance Time (s)	Swing Time (s)	Stride Length (m)	Walking Speed (m/s)
Elderly (Health)	STW	100.08 ± 3.79	1.18 ± 0.07	64.19 ± 1.52	35.81 ± 1.52	0.76 ± 0.06	0.42 ± 0.02	1.21 ± 0.17	1.04 ± 0.16
	VFT	84.27 ± 7.10	1.44 ± 0.13	68.40 ± 1.44	31.60 ± 1.44	0.98 ± 0.10	0.45 ± 0.04	1.02 ± 0.14	0.72 ± 0.12
	STW/VFT	0.000 *	0.000 *	0.000 *	0.000 *	0.000 *	0.000 *	0.000 *	0.000 *
Elderly (MCI)	STW	96.47 ± 5.78	1.22 ± 0.07	65.64 ± 2.24	34.36 ± 2.24	0.80 ± 0.07	0.42 ± 0.02	1.15 ± 0.14	0.94 ± 0.14
	VFT	78.37 ± 8.40	1.55 ± 0.21	69.47 ± 2.16	30.53 ± 2.16	1.08 ± 0.15	0.47 ± 0.06	0.96 ± 0.16	0.63 ± 0.10
	STW/VFT	0.000 *	0.002 *	0.000 *	0.000 *	0.002 *	0.003 *	0.000 *	0.000 *
Elderly (PD)	STW	96.23 ± 6.76	1.20 ± 0.10	65.00 ± 1.98	35.00 ± 1.98	0.78 ± 0.09	0.42 ± 0.02	1.00 ± 0.13	0.84 ± 0.15
	VFT	77.63 ± 13.88	1.57 ± 0.39	69.63 ± 3.32	30.37 ± 3.32	1.10 ± 0.34	0.47 ± 0.06	0.86 ± 0.15	0.58 ± 0.18
	STW/VFT	0.000 *	0.003 *	0.000 *	0.000 *	0.003 *	0.015 *	0.000 *	0.000 *
CSVD	STW	93.97 ± 8.98	1.29 ± 0.12	67.72 ± 2.65	32.28 ± 2.65	0.87 ± 0.10	0.42 ± 0.04	0.85 ± 0.17	0.67 ± 0.16
	VFT	80.18 ± 10.85	1.53 ± 0.22	72.19 ± 3.257	27.81 ± 3.26	1.11 ± 0.191	0.42 ± 0.05	0.66 ± 0.17	0.44 ± 0.15
	PTW	85.87 ± 11.46	1.41 ± 0.18	70.43 ± 3.48	29.57 ± 3.48	1.00 ± 0.16	0.41 ± 0.05	0.72 ± 0.21	0.52 ± 0.18
	STW/VFT	0.000 *	0.000 *	0.000 *	0.000 *	0.000 *	0.532	0.000 *	0.000 *
	STW/PTW	0.000 *	0.000 *	0.000 *	0.000 *	0.000 *	0.779	0.000 *	0.000 *

* *p* < 0.05.

## Data Availability

Due to the nature of this research, the participants of this study did not agree for their data to be shared publicly, so supporting data are not available.
